# Synthesis, characterization, and properties of a novel aromatic ester-based polybenzoxazine

**DOI:** 10.1039/c9ra10191h

**Published:** 2020-02-20

**Authors:** Chunli Zhu, Xing Gao, Weixi Fan, Xiaofen Fu

**Affiliations:** School of Chemistry and Biological Engineering, Qilu Institute of Technology Jinan 250200 P. R. China i40597087qiaoqia3@163.com

## Abstract

Polybenzoxazines with molecular design flexibility have excellent properties by using suitable raw materials. A new benzoxazine monomer terephthalic acid bis-[2-(6-methyl-4*H*-benzo[*e*][1,3]oxazin-3-yl)]ethyl ester (TMBE) with bis-ester groups has been synthesized from the simple esterification reaction of terephthaloyl chloride and 2-(6-methyl-4*H*-benzo[*e*][1,3]oxazin-3-yl)-ethanol (MB-OH). The chemical structure of TMBE was characterized by Fourier transform infrared spectroscopy (FT-IR) and nuclear magnetic resonance spectroscopy (^1^H-NMR, ^13^C-NMR). Polymerization behavior of TMBE was studied by differential scanning calorimetry (DSC) and FT-IR after each cure stage. The cross-linked polybenzoxazine (PTMBE) gave a transparent film through the thermal casting method. The dynamic mechanical analysis of PTMBE showed that the *T*_g_ was 110 °C. Thermogravimetric analysis reveals better thermal stability as evidenced by the 5% and 10% weight-loss temperatures (*T*_d5_ and *T*_d10_) of PTMBE, which were 263 and 289 °C, respectively, with a char yield of 27% at 800 °C. The tensile test of the film revealed that the elongation at break was up to 14.2%.

## Abstract 2

A novel benzoxazine monomer contain ester group was obtained by an indirect molecular design method. Its polymer showed excellent flexibility.

## Graphical abstract

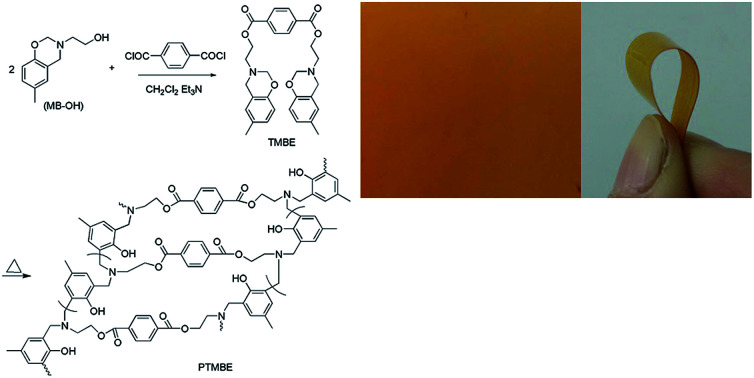

## 1. Introduction

As a new phenolic-type resin, polybenzoxazines possess attractive properties, such as high glass transition temperature,^1,2^ flame retardancy,^3^ low water absorption,^4^ and low cost.^5^ They are applied to the field of halogen-free flame retardant laminates, vacuum pump rotors, printed circuit boards, friction materials, composites, and other fields.^6,7^ Nevertheless, inherent brittleness of polybenzoxazines, especially in aromatic polybenzoxazines, limits their further development in the chemical industry.

There are generally three reported approaches to improve the toughness of polybenzoxazines. The first approach is introducing rubber,^8,9^ the flexible heat-resistant linear polymer^10–14^ into resins as the toughening agent to improve toughness. The second approach is designing inherently tough benzoxazine monomers containing soft segments to afford tough polybenzoxazines.^15,16^ The third approach is the toughening of polybenzoxazines by inorganic nanoparticles.^17–19^ Among them, the designing of inherently tough benzoxazine monomers and polybenzoxazines prepolymers was a significant approach to improve the flexibility of polybenzoxazines from the molecular level, and to increase the degree of chemical crosslinking between the two phases.

The tough benzoxazine monomers were synthesized, including monofunctional and difunctional benzoxazines from available phenols containing flexible groups, primary amines and formaldehyde.^4,20–23^ These monomers improve the flexibility of polybenzoxazines with dramatically scarifying their heat resistance. Therefore, some main-chain type polybenzoxazines^24,25^ were synthesized by Mannich reaction with polyether diamines of different molecular weights or PDMS with different molecular weights for the diamine terminal, bisphenol A, and formaldehyde. Such high-molecular-weight prepolymers exhibited significantly improved toughness due to the soft segments and generated a high cross-linking degree due to the polybenzoxazine component.

The other approach to improve toughness is indirectly preparing of polybenzoxazine prepolymers using polyesterification,^14^ coupling reactions, alternating copolymerization of donor–acceptor monomers,^26^ Diels–Alder reaction,^27^ polyetherification,^28^ hydrosilylation^29^ These synthetic approaches also necessarily yield high-performance polybenzoxazines with improved toughness. The novel polyesters were synthesized from the polycondensation reaction of bisbenzoxazine-diol, pyromellitic dianhydride, and 4,4′-(hexafluoroisopropylidene)diphthalic anhydride.^14^ The molecular weights of polyesters were in the range of 5800–7000 Da with benzoxazine moieties in the main chain exhibited high flexibility induced by the soft pentyl and esters groups and comparable thermal stability concerning low molar mass analogous. Aydogan^29^ prepared polysiloxanes containing benzoxazine units in the main chain by hydrosilylation of 1,1,3,3-tetramethyldisiloxane and diallyl functional benzoxazines. To further improve its flexibility, polysiloxanes chain was extended by the reaction of poly (bisbenzoxazinedimethylsiloxane)s and octamethyl-cyclotetrasiloxane in the presence of tetrabutylammonium hydroxide as a catalyst.

Based on this idea, our work presents a facile route for synthesizing high-purity aromatic benzoxazine containing bis-ester groups. First, 2-(6-methyl-4*H*-benzo[*e*][1,3]oxazin-3-yl)-ethanol (MB-OH) was synthesized from formaldehyde solution (37%), ethanolamine, and *p*-cresol. Then, terephthalic acid bis-[2-(6-methyl-4*H*-benzo[*e*][1,3]oxazin-3-yl)]ethyl ester (TMBE) with bis-ester groups has been synthesized from the simple esterification reaction of MB-OH and terephthaloyl chloride. The crosslinked polybenzoxazine (PTMBE) gave transparent flexible film through the thermal casting method. The thermal and mechanical properties of aromatic ester-based polybenzoxazine were studied and compared with respect to polybenzoxazines analogous.

## 2. Material and methods

### 2.1. Materials

Potassium hydroxide, *p*-cresol (98%) were purchased from Sinopharm Chemical Reagent Co., Ltd. 2-Aminoethanol, dioxane, triethylamine (Et_3_N), methylene chloride was obtained from Tianjin Fu Jin Fine Chemical Co., Ltd. Formaldehyde (37 wt% in water), terephthaloyl chloride, *n*-hexane was used as received. Methylene chloride used after distillation.

### 2.2. Synthesis of MB-OH

MB-OH was synthesized according to the literature.^12^ A suspension of paraformaldehyde (15.3 mL, 210 mmol), 30 mL 1,4-dioxane were added to a 250 mL three-neck flask in an ice bath. Then 2-aminoethanol (6.0 mL, 100 mmol) was added drop-wise to the system. The mixture stirred at room temperature for about 30 min. After adding *p*-cresol (10.6 mL, 100 mmol), the temperature was gradually increased to 70 °C, and stirring was continued for another 3.5 h. A yellowish viscous fluid was obtained after removing the solvent through a rotary evaporator. Then the viscous liquid was dissolved in dichloromethane, washed with 1 L of 0.1 N aqueous KOH and finally three times with 1 L of distilled water. The methylene chloride solution was dried with MgSO_4_, filtered, and concentrated under vacuum to afford crude product. The crude product was recrystallized with *n*-hexane to give pure white solid of MB-OH (9.53 g, 51%).

^1^H NMR (CDCl_3_, 300 MHz, *δ*): 3.98 ppm (s, Ar–*CH_2_–N), 4.85 ppm (s, O–*CH_2_–N), 2.95 ppm (t, N–*CH_2_–C), 3.69 ppm (t, C–*CH_2_–O), 2.24 ppm (s, Ph–*CH_3_).

FTIR (KBr) *ν*: 1034 cm^−1^ (C–O–C symmetric stretching), 1232 cm^−1^ (C–O–C asymmetric stretching), 1502 cm^−1^ (trisubstituted benzene ring), 940 cm^−1^ (oxazine ring).

### 2.3. Synthesis of TMBE

Into a 100 mL dry round-bottom flask equipped with a calcium chloride drying tube, 20 mL dry methylene chloride, MB-OH (1.9 g, 10 mmol), triethylamine (1.7 mL, 1.4 mmol) were added and mixed at room temperature for about 30 min. Then terephthaloyl chloride (1.0 mL, 5.0 mmol) was added at 5 °C. The mixtures were stirred at room temperature for 2 d and kept refluxing for 4 h. A yellow transparent solution was obtained after cooling to room temperature. The solution was washed with 1 L of 0.5 N aqueous NaOH and finally three times with 1 L of distilled water. The methylene chloride solution was dried with MgSO_4_, filtered, and concentrated under vacuum to afford crude product (1.6 g, yield: 61%). The product was purified by chromatography (v_methylene chloride_ : v_ethyl acetate_ = 2 : 1) to afford white power solid.

^1^H NMR (CDCl_3_, 300 MHz, *δ*): 4.05 ppm (s, Ar–*CH_2_–N), 4.88 ppm (s, O–*CH_2_–N), 3.18 ppm (t, N–*CH_2_–C), 4.52 ppm (t, C–*CH_2_–O), 2.27 ppm (s, Ph–*CH_3_).

^13^C NMR (CDCl_3_, 300 MHz, *δ*): 50.70 ppm (Ar–*CH_2_–N), 82.85 ppm (O–*CH_2_–N), 50.38 ppm (N–*CH_2_–C), 63.65 ppm (C–*CH_2_–O), 18.30 ppm (Ph–*CH_3_), 165.71 ppm (O–C*=O).

FTIR (KBr) *ν*: 1019 cm^−1^ (C–O–C symmetric stretching), 1277 cm^−1^ (C–O–C asymmetric stretching), 1506 cm^−1^ (trisubstituted benzene ring), 932 cm^−1^ (oxazine ring), 1720 cm^−1^ (C=O stretching).

### 2.4. Polymerization of TMBE

The amount of TMBE was melted, stirred and transferred to a rectangular aluminum foil mold and then cured at 120 °C/2 h, 140 °C/2 h, 160 °C/2 h, 180 °C/2.5 h in an air-circulating oven. After that, samples were allowed to cool slowly to room temperature to be tested.

### 2.5. Characterizations

FTIR spectra were obtained with Bruker Tensor 27 FTIR spectrometer (Bruker, GM) in which samples were preparing as KBr pellets. ^1^H-NMR and ^13^C-NMR spectra were recorded on a Bruker Avance 300 instrument (Bruker) using CDCl_3_ as the solvent and TMS as the internal standard. Differential scanning calorimetry (DSC) was conducted using a DSC SP instrument (Rheometric Scientific) at a heating rate of 10 °C min^−1^ under nitrogen. Thermogravimetric analysis (TGA) was performed on a TGA/DSC STARe System instrument (Mettler-Toledo) at a heating rate of 10 °C min^−1^ under N_2_ atmosphere. The gas flow rate was 100 mL min^−1^. The dynamic mechanical analysis (DMA) of samples was carried out using a Mettler-Toledo DMA/SDTA861e instrument (Mettler-Toledo). The specimen with dimensions of approximately 3.00 × 2.20 × 0.8 mm^3^ was tested by a shear mode at 1 Hz in the temperature range of 30–200 °C at a heating rate of 3 °C min^−1^. Tensile measurement were also recorded with Material Testing Machine Model UTM5105 at a crosshead speed of 1 mm min^−1^. Each sample with a dimension of approximately 20.0 × 5.0 × 1.0 mm^3^ was tested from an average of at least 5 tests.

## 3. Results and discussion

### 3.1. Preparation of TMBE and its polymer

**Scheme 1:**
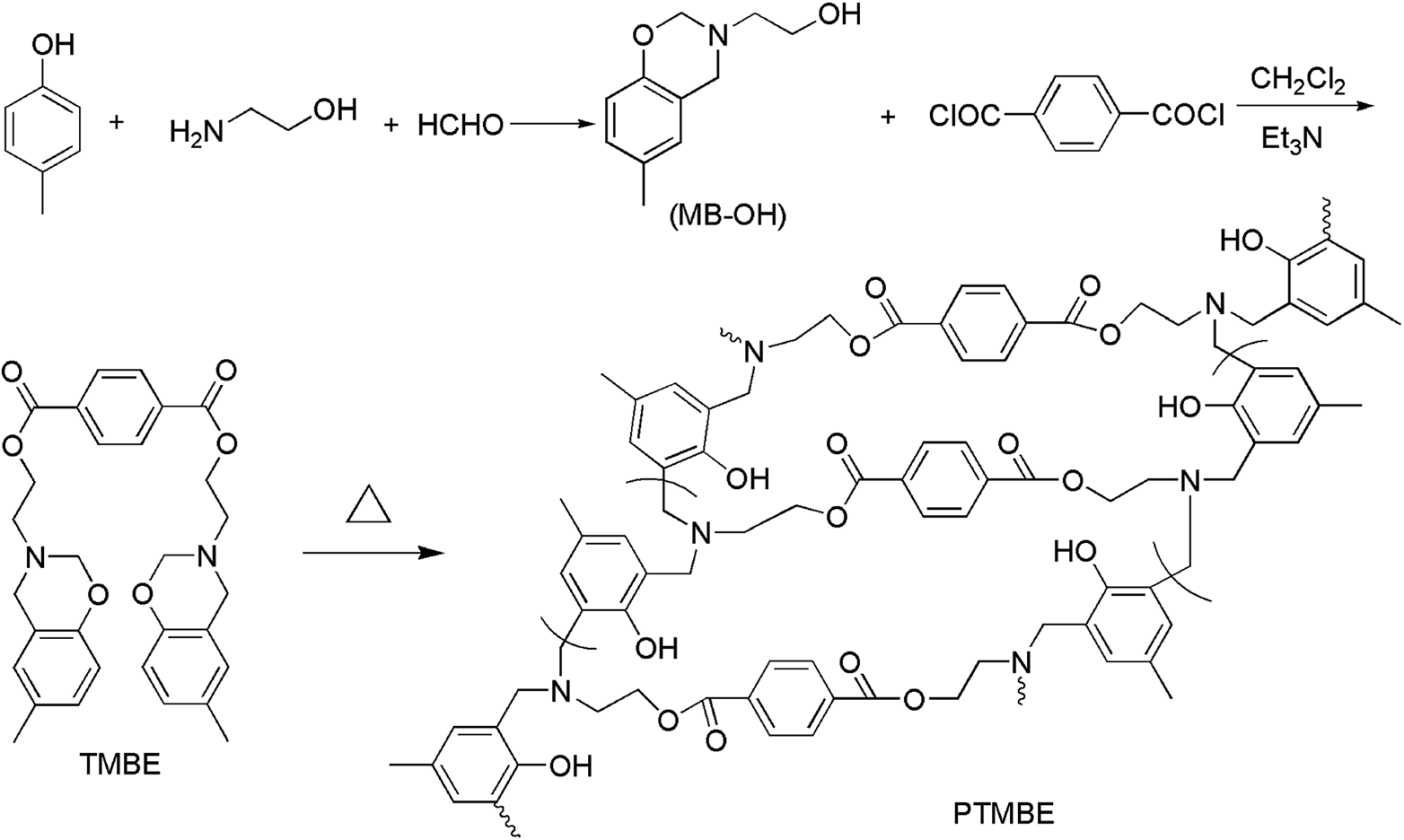
Synthetic route to TMBE and PTMBE.

Scheme 1 illustrates our strategy for the preparation of TMBE and PTMBE. First, according to reference, we synthesized containing hydroxyl benzoxazine (MB-OH). Second, using triethylamine as deacid reagent, dry dichloromethane as solvent, TMBE was obtained *via* the esterification reaction of MB-OH and terephthaloyl chloride at a molar ratio of 2 : 1. TMBE was easily soluble in common low-boiling organic reagents, such as dichloromethane, chloroform, and tetrahydrofuran. Finally, PTMBE was formed by the cross-linking of TMBE under thermal curing reaction with polymerization temperature of 180 °C.

### 3.2. Characterization of TMBE

**Fig. 1:**
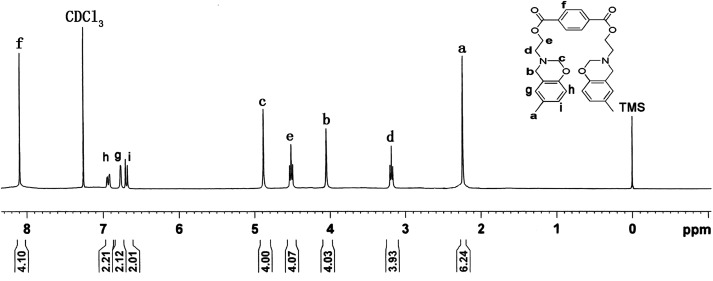
^1^H-NMR spectrum of TMBE.

The chemical structure of TMBE was confirmed by NMR and FT-IR spectral analysis. As can be seen from Fig. 1 where the ^1^H NMR spectrum of TMBE is presented, the appearance of the protons resonating at 4.05 ppm (–O–*CH_2_–N) and 4.88 ppm (Ar–*CH_2_–N) was clear evidence for the benzoxazine ring formation on TMBE. Moreover, the peaks at 3.18 ppm and 4.52 ppm assigned to protons of –N–*CH_2_–C– and C–*CH_2_–O– were observed, respectively. The integral ration of these peaks was close to the theoretical ratio of 2 : 2 : 2 : 2. The signal peak appeared at 2.27 ppm corresponding to the Ph–*CH_3_. The peak appeared at 8.10 ppm corresponding to the protons of the benzene ring linked to the ester group. The peaks appeared at 6.68 ppm, 6.75 ppm, and 6.95 ppm assigned to protons of benzene ring linked to the oxazine ring.

**Fig. 2:**
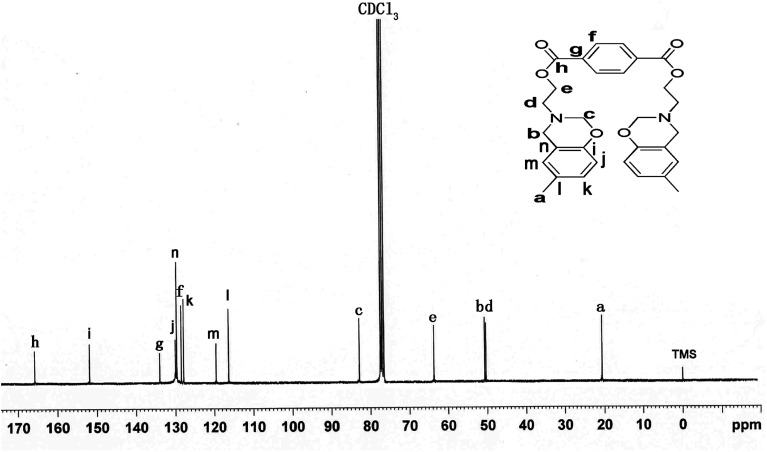
^13^C-NMR spectrum of TMBE.

The ^13^C NMR spectrum presented from Fig. 2 further proved the structure of TMBE. The carbon resonances of the oxazine ring at 50.7 ppm for Ar–*CH_2_–N and at 82.9 ppm for O–*CH_2_–N were clearly observed, respectively. The carbon resonances at 50.38 and 63.65 ppm of N–*CH_2_–C and C–*CH_2_–O were clearly appeared, respectively. The peak at 165.71 and 20.30 ppm were characteristic absorptions of O–C*=O and Ph–*CH_3_, respectively. The resonance at 128.46 and 133.96 ppm corresponded to carbon atoms in the aromatic ring of terephthalate. Others were characteristic absorptions attributed to the aromatic ring of benzoxazine.

**Fig. 3:**
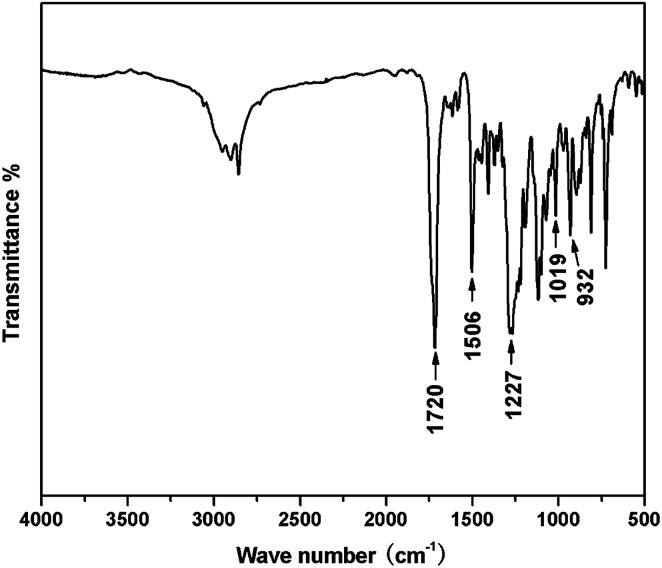
FTIR spectrum of the TMBE.

FT-IR spectra also give evidence for the formation of benzoxazine containing bis-ester groups (Fig. 3). The characteristic peak at 932 cm^−1^ due to the C–H out-of-plane vibration in the benzene ring where an oxazine ring attached was clearly observed. Additionally, the strong peak at 1720 cm^−1^ was characteristic absorption of the carbonyl in terephthalate.

### 3.3. Polymerization of TMBE

**Fig. 4:**
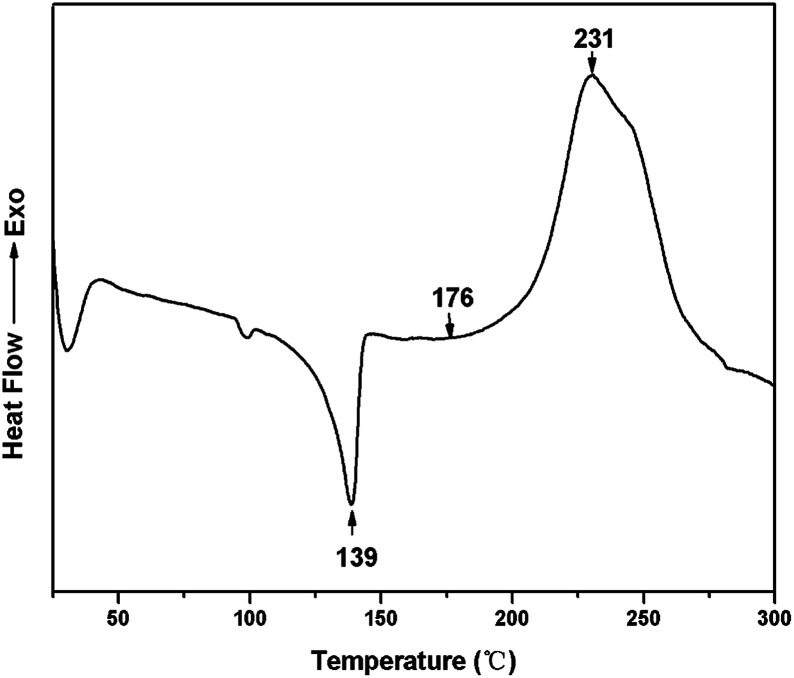
DSC thermogram of TMBE.

The DSC heating-scan thermogram of TMBE was depicted in Fig. 4. The sharp endothermic peak appearing at 139 °C corresponds to the melting temperature (*T*_m_) of TMBE. TMBE showed unimodal curing behaviour as observed from the single exothermic peak, which is from the ring-opening polymerization of the benzoxazine ring. The onset and peak top temperatures of the peak are 176 and 231 °C, respectively. The polymerization temperature of TMBE is similar to the values reported to other traditional benzoxazine compounds.^30^ DSC result of curing TMBE show the presence of aliphatic chains (C4) in the benzoxazine structure has little effect on the polymerization behavior of TMBE and only a lower temperature is required for polymerization.

**Fig. 5:**
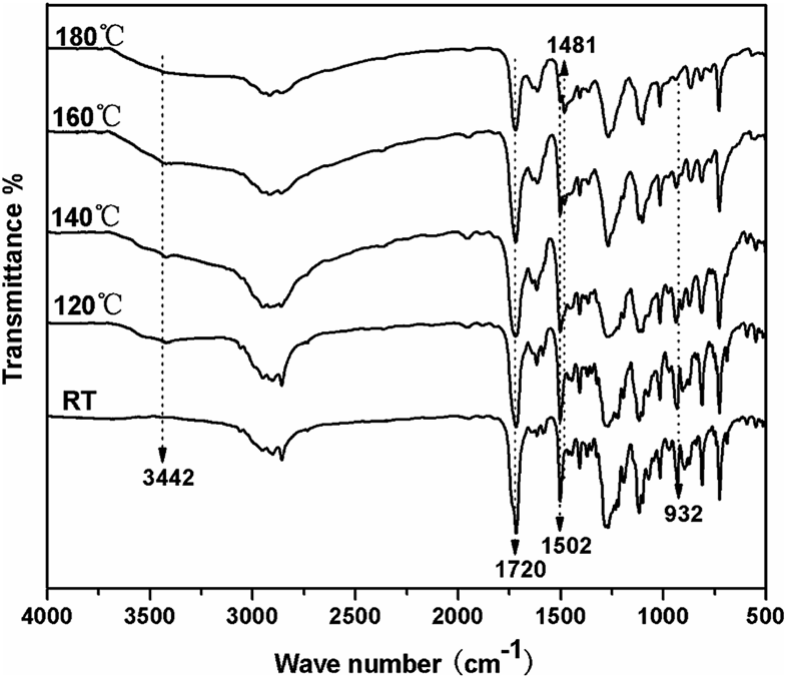
FTIR spectra of TMBE after curing at each temperature.

The polymerization behavior of TMBE was also monitored by FTIR after curing at each temperature (Fig. 5). With the increase of temperature, the intensity of the characteristic absorption peak at 932 cm^−1^ due to benzoxazine structure gradually decreased due to the opening of the oxazine ring and disappeared after the 180 °C for 2.5 h. The intensity of absorption bands at 1502 cm^−1^ due to the trisubstituted benzene ring (stretching) also decreased. Meanwhile, some new absorption bands appeared at 3442 cm^−1^ due to the phenolic hydroxy and at 1481 cm^−1^ due to the tetrasubstituted benzene ring, suggesting that the ring-opening polymerization of TMBE occurred. Both DSC and IR spectroscopy indicates the formation of polybenzoxazine by polymerization at 180 °C.

### 3.4. Thermal property of PTMBE

**Fig. 6:**
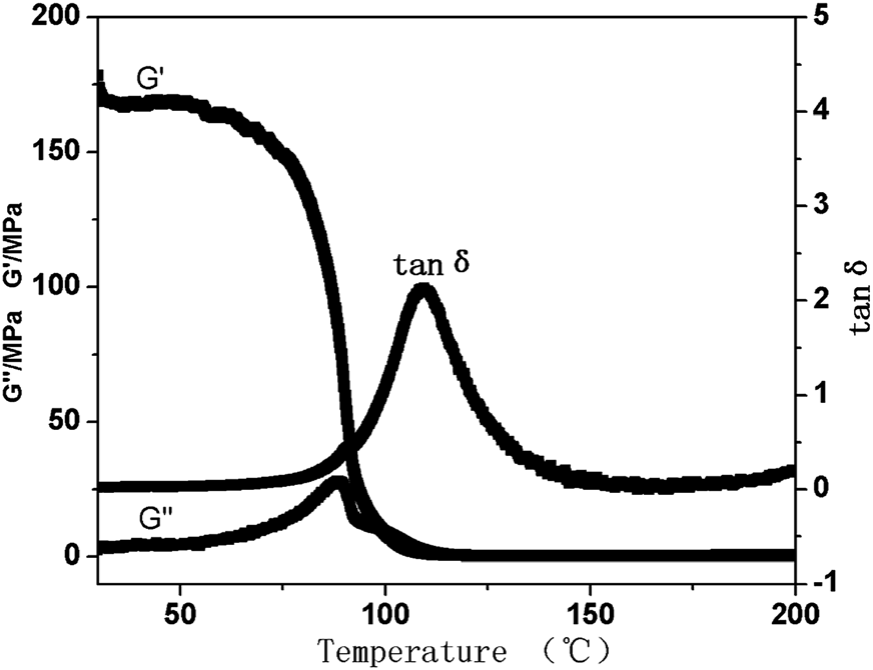
DMA curves of PTMBE.

Dynamic mechanical analysis (DMA) was performed to study the viscoelastic properties of PTMBE (Fig. 6). At 30 and 80 °C, the storage modulus (*G*′) values are 168 and 100 MPa, respectively, whereas the loss modulus (*G*′′) values are 11 and 27 MPa, respectively. For poly(BA-a) (bisphenol-A and aniline-based polybenzoxazine), the storage modulus *G*′ of 1.9 × 10^3^ MPa is higher than that of PTMBE due to their high aromatic content. It has been clearly demonstrated from DMA that the flexibility of PTMBE is better than poly(BA-a). The *T*_g_ was observed at 110 and 89 °C from the maximum loss factor and maximum loss modulus, respectively. The decrease of *T*_g_ can be attributed to the flexible alkyl chain in PTMBE because of a lower energy barrier for motion.

**Fig. 7:**
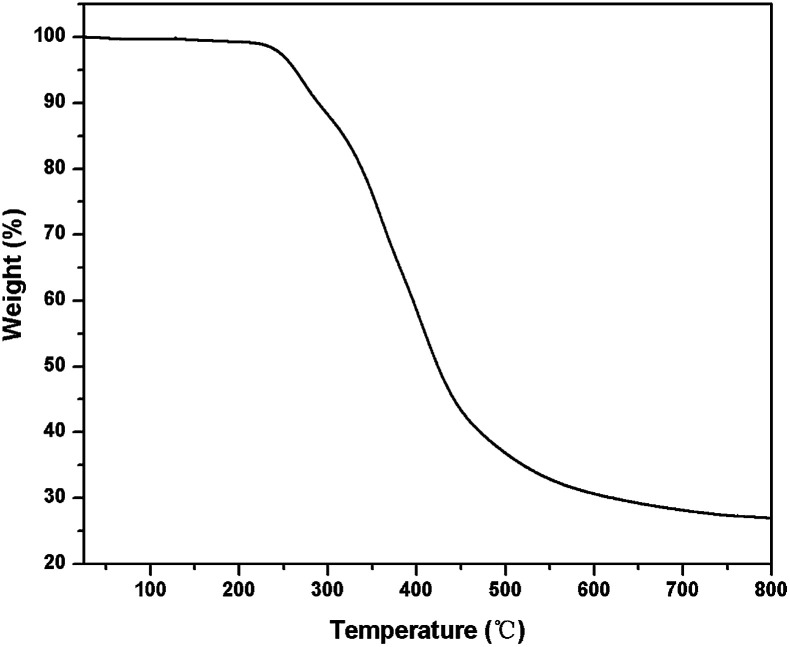
TGA curve of PTMBE.

TGA was performed to determine the thermal stability of PTMBE. Fig. 7 shows the TGA thermograms of PTMBE. In nitrogen, 5 and 10% weight loss temperatures (*T*_d5_ and *T*_d10_) of PTMBE were 263 and 289 °C, respectively. The char yield of PTMBE was up to 27.0% at 800 °C. The *T*_d5_ and *T*_d10_ of PTMBE were decreased by about 50 °C compared with poly(BA-a). The char yield of PTMBE at 800 °C has a slight drop compared with poly(BA-a). PTMBE shows the lower thermal stability than poly(BA-a) attributed as mentioned previously to the flexible aliphatic chain.

### 3.5. Mechanical properties of PTMBE

**Fig. 8:**
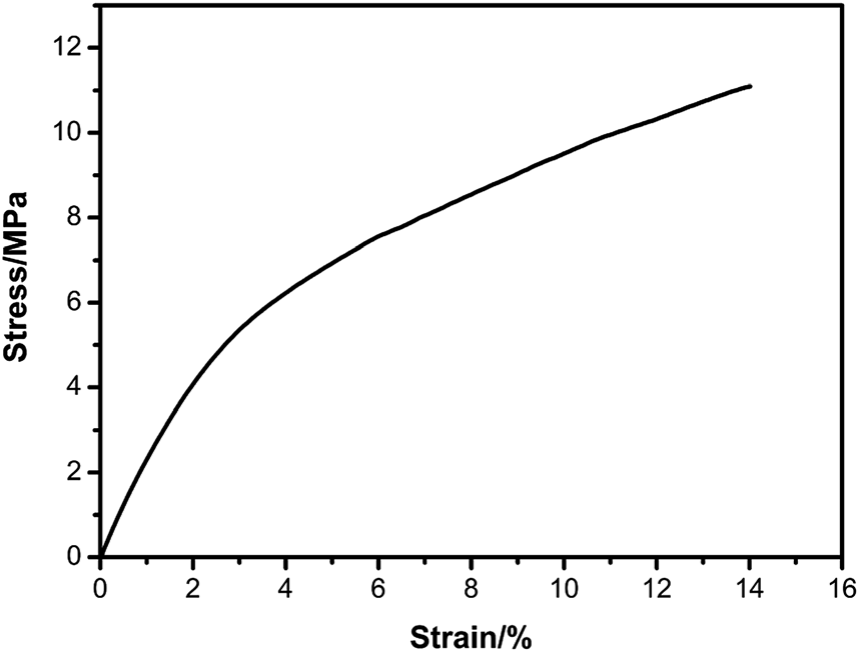
Stress–strain curve of PTMBE.

The stress–strain curve was showed to study the mechanical property for the PTMBE film (Fig. 8). The PTMBE film showed elongation at a break of 14.2% and the tensile strength of 11.3 MPa. It is reported that the PB-a film is very brittle with elongation at break of 1.3%.^30^ The elongation at break of PTMBE was larger than that of PB-a. Namely, PTMBE had remarkably improved toughness, and the film is easy to bend. Interestingly, in spite of the introduction of the aromatic ester group, PTMBE still showed the high elongation at break comparing with polybenzoxazines^20^ only containing linear long aliphatic chains (C12). The toughness enhancement of PTMBE is attributed to the long flexible alkyl chains, which enhances the mobility of segments under load and thereby increases the ultimate elongation. The tensile modulus of PTMBE is 248.0 MPa, lower than that of PBA-a (4.3 GPa) with rigid backbone.^30^ Therefore, the incorporation of the long aliphatic chain and aromatic ester group into PTMBE network structure gave flexible polymer film without excessively scarifying its heat resistance.

**Fig. 9:**
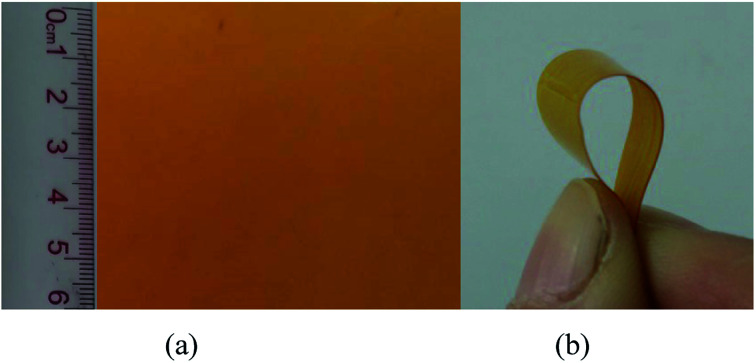
Appearance of PTMBE film (a) and (b).

A yellowish PTMBE film (7 × 6 cm^2^) with 150–200 μm in thickness was obtained through the thermal casting method in Fig. 9(a). A larger-sized film (20 × 20 cm^2^) was also prepared in our lab according to the same procedure. Besides, the PTMBE film had remarkably improved toughness, and is easy to bend in Fig. 9(b).

## 4. Conclusions

By utilizing a clean and facile route, we have prepared a high-purity aromatic ester-based benzoxazine monomer and its polymer. Thermally activated polymerization of benzoxazine monomer provided flexible, uniform polymer film. The novel polybenzoxazine film exhibited significantly improved toughness due to the long aliphatic chain. The storage modulus and glass transition temperature of PTMBE were 168 MPa and 110 °C, respectively. Regarding thermal stability, PTMBE showed lower thermal stability than poly(BA-a) due to the aliphatic hydrocarbon chain. It is anticipated to find applications of PTMBE as a self-healing material.

## Conflicts of interest

There are no conflicts to declare.

## Supplementary Material
